# Middlesex Hospital

**Published:** 1902-06-14

**Authors:** 


### MIDDLESEX HOSPITAL.

### QUARTERLY COURT.

On Thursday, May 29th, Lord Lathom presided over the Quarterly Court of Governors of the Middlesex Hospital. Twenty-seven were present.

Lord Sandhurst proposed the adoption of the quarterly report, and commented on the retirement of Sir Ralph Thompson, K.C.B., from the position of chairman; he had not, however, vacated his seat on the board. Lord Sandhurst said the weekly board was most anxious to do everything in its power for the furtherance of science and the good of the patients, relying always on the opinion of the Medical Committee. The chairman of that committee was present, and he hoped he would have something to say about the new clinical laboratory which the court was asked to approve.

Dr. Pasteur, chairman of the Medical Committee, said the clinical laboratories were a considerable extension of the bacteriological department, the only fault of which was that it was too limited. Extensive microscopic experiments were needed, and it was for these that the new department was to be opened.

Mr. Burnand, treasurer, announced that the contents of the collecting boxes amounted to £19 5s. 9d., and the collecting clerk stated that he had received £1,542 5s. 2d.

The other proceedings were brief and purely formal, and the court then became special, to sanction the sale of stock for the realisation of £20,000 during the year.

Notes on the Report.

The old "Laffan" Ward has been converted into a special operating theatre for gynæcological cases in connection with "Prudhoe" Ward, to which it is adjacent, at a cost of £779 18s. The courtyard has been re-asphalted, after a lapse of 16 years, on the advice of the architect. The yearly cleaning will be done this year without employing a contractor. The laundry at Hendon is approaching completion, and will soon be ready for use; a further outlay of £1,094 16s. has been found necessary for the erection of housing accommodation for the head laundress and the engineer, an extension of the roadway, and the fencing in of the property. The building of the extension to the Nursing Home is proceeding, and it is hoped that the end of the year will see it completed. Dr. Frederick Hetley, a former student of the Medical School, has since 1884 given an annual prize of the value of £25 for the best results of an oral examination in medicine, surgery, and obstetric medicine; he has recently endowed this prize by a donation of £1,000, any balance of the interest to be devoted to the Cancer Fund. The report records his death with regret.

The New Clinical Laboratory.

By completely rearranging and refitting the existing pathological laboratory and the room at present occupied by the lecturer on pathology, sufficient accommodation has been found to provide departments for chemical analysis, bacteriological investigation, and microscopical examination of morbid material.

The cost of such conversion and fitting, including an approximate cost of instruments and appliances, is estimated at £547.

The annual expenditure for the salaries of the proposed staff, and for the upkeep of the laboratory, is estimated at £1,480, as follows: A director, to perform the duties of directing the clinical laboratory conjointly with those in connection with the cancer laboratories, at a minimum salary of £500; two assistants, with salaries of £200 and £100 per annum respectively; two laboratory assistants at 12s. per week each, £63; upkeep estimated at £70 per annum. The salaries of the bacteriologist and his assistant being merged in this scheme will effect a saving of £180. The total approximate estimate for the first year will therefore amount to £1,300.

CANCER RESEARCH.

The first volume of "Reports from the Cancer Research Laboratories" is now going through the press, and will shortly be issued. The staff of the laboratories are now engaged on a second volume of the reports, which the director hopes to have ready for the press before the end of the year.

The board have at present under their consideration recommendations from the Cancer Investigation Committee as to the provision of additional laboratory accommodation, and an increase in the present staff of the laboratories. The present arrangements will not allow full use to be made of the large quantity of material available for investigation, and extra facilities are urgently needed.

In view of the great and increasing interest which is now being taken in questions relating to the causation of cancer, says the report, it may be hoped that this work, for which the Middlesex Hospital provides almost unique opportunities, will not be kept back for want of funds.

In addition to the X-ray apparatus, which has been in use for treatment in the cancer wards for some three years past, apparatus has now been fitted up for carrying out a new form of treatment by means of what are known as "high frequency" currents.

